# Supplementary material for: Effects of Escherichia coli LPS Structure on Antibacterial and Anti-Endotoxin Activities of Host Defense Peptides
Source: Pharmaceuticals (Basel). 2023 Oct 18;16(10):1485. doi: 10.3390/ph16101485 (PMC10609994; doi:10.3390/ph16101485)
Supplement: Supplementary file 1 [file pharmaceuticals-16-01485-s001.zip › pharmaceuticals-2640787-supplementary.pdf]

Supplementary data

Effects of *Escherichia. coli* LPS structure on antibacterial and anti-endotoxin activities of host defense peptides

Ali Javed 1,2,†, Melanie D. Balhuizen 3,†, Arianne Pannekoek 1, Floris J. Bikker 4, Dani A. C. Heesterbeek 5, Henk P. Haagsman 3, Femke Broere 1, Markus Weingarth 2 and Edwin J. A. Veldhuizen 1,*

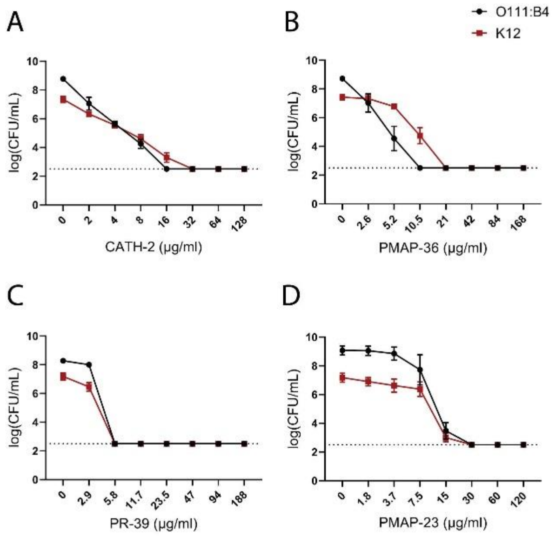

Figure S1. Determination of MBC values (µg/ml) of HDPs against *E. coli* strains (O111:B4 or K-12). MBC values of CATH-2 **(A),** PMAP-36 **(B),** PR-39 **(C),** and PMAP-23 **(D)** were determined by colony count assay. Surviving bacterial colonies were detected after incubation with HDPs for 3 h in MHB. Shown are mean ± SEM of three independent experiments, dashed line shows the detection limit of the assay.

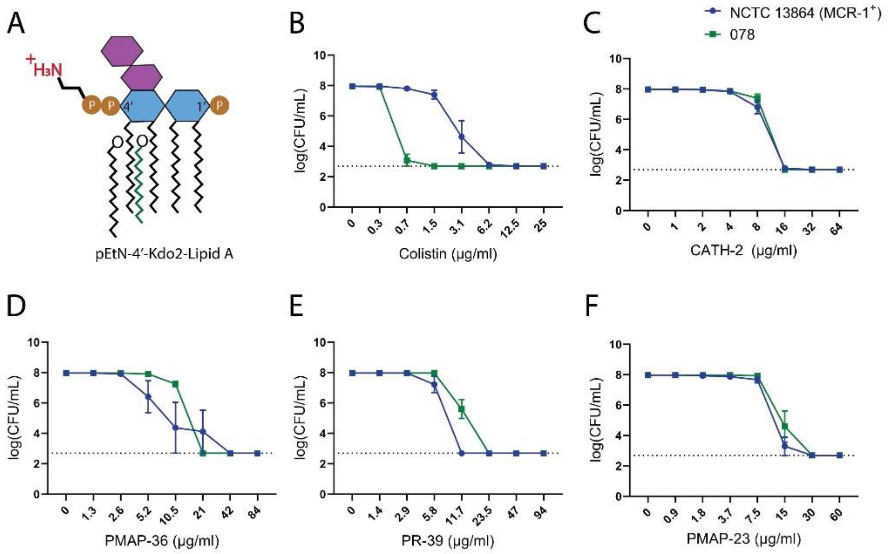

Figure S2. Determination of MBC values (μg/ml) of HDPs against MCR-1+ *E. coli*. **(A)** MCR-1+ bacteria have a Phosphoethanolamine (pEtN) group attached to 1′or 4′-Phosphate of Lipid A in the LPS. MBC values of Colistin **(B)**, CATH-2 **(C)**, PMAP-36 **(D)**, PR-39 **(E)** and PMAP-23 **(F)** for E. coli strains (NCTC 13864 or 078) were determined by colony count assay. Surviving bacterial colonies were detected after incubation with HDPs for 3 h in MHB. Shown are mean ± SEM of three independent experiments, dashed line shows the detection limit of the assay.

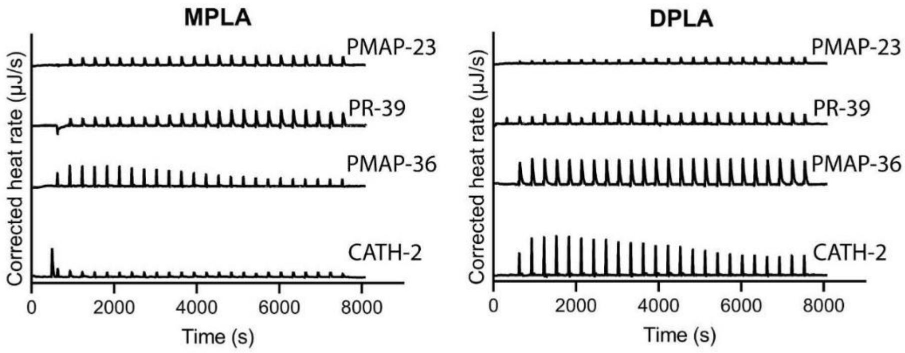

Figure S3. ITC spectra of the interaction between Lipid A species and peptides. 200 μM of CATH-2, PMAP-36, PR-39 or PMAP-23 was titrated into *E. coli* Lipid A (MPLA or DPLA) solution (25μM) and heat rates were recorded. Shown is a representative of two measurements.

Table S1. Dissociation constants and stoichiometry calculated using ITC data for interactions between *E. coli* LPS or Lipid A and HDPs. Data was fitted using an independent model, except for PMAP-36 and *E. coli* O111 LPS where a multiple sites model was used. n.d. = not defined which indicates no binding occurred. Shown is an average of two experiments.

	***E. coli* O111**		***E. coli* K-12**		***MPLA***		***DPLA***	
	**Kd**	**n**	**Kd**	**n**	**Kd**	**n**	**Kd**	**n**
**CATH-2**	2.55E-06	0.66	1.15E-06	0.35	n.d.	n.d.	1.02E-06	1.5
**PMAP-36**	2.50E-08<br>6.54E-08	0.11<br>1.56	n.d.	n.d.	2.09E-06	1.3	n.d.	n.d.
**PR-39**	3.68E-07	0.34	n.d.	n.d.	n.d.	n.d.	n.d.	n.d.
**PMAP-23**	1.83E-06	1.18	3.35E-07	1.16	n.d.	n.d.	n.d.	n.d.
